# Comprehensive Brain MRI Segmentation in High Risk Preterm Newborns

**DOI:** 10.1371/journal.pone.0013874

**Published:** 2010-11-08

**Authors:** Xintian Yu, Yanjie Zhang, Robert E. Lasky, Sushmita Datta, Nehal A. Parikh, Ponnada A. Narayana

**Affiliations:** 1 Division of Neonatal-Perinatal Medicine, Department of Pediatrics, University of Texas Health Science Center at Houston Medical School, Houston, Texas, United States of America; 2 Center for Clinical Research and Evidence Based Medicine, University of Texas Health Science Center at Houston Medical School, Houston, Texas, United States of America; 3 Department of Diagnostic and Interventional Imaging, University of Texas Health Science Center at Houston Medical School, Houston, Texas, United States of America; Cornell University, United States of America

## Abstract

Most extremely preterm newborns exhibit cerebral atrophy/growth disturbances and white matter signal abnormalities on MRI at term-equivalent age. MRI brain volumes could serve as biomarkers for evaluating the effects of neonatal intensive care and predicting neurodevelopmental outcomes. This requires detailed, accurate, and reliable brain MRI segmentation methods. We describe our efforts to develop such methods in high risk newborns using a combination of manual and automated segmentation tools. After intensive efforts to accurately define structural boundaries, two trained raters independently performed manual segmentation of nine subcortical structures using axial T2-weighted MRI scans from 20 randomly selected extremely preterm infants. All scans were re-segmented by both raters to assess reliability. High intra-rater reliability was achieved, as assessed by repeatability and intra-class correlation coefficients (ICC range: 0.97 to 0.99) for all manually segmented regions. Inter-rater reliability was slightly lower (ICC range: 0.93 to 0.99). A semi-automated segmentation approach was developed that combined the parametric strengths of the Hidden Markov Random Field Expectation Maximization algorithm with non-parametric Parzen window classifier resulting in accurate white matter, gray matter, and CSF segmentation. Final manual correction of misclassification errors improved accuracy (similarity index range: 0.87 to 0.89) and facilitated objective quantification of white matter signal abnormalities. The semi-automated and manual methods were seamlessly integrated to generate full brain segmentation within two hours. This comprehensive approach can facilitate the evaluation of large cohorts to rigorously evaluate the utility of regional brain volumes as biomarkers of neonatal care and surrogate endpoints for neurodevelopmental outcomes.

## Introduction

More than 50% of extremely low birth weight (ELBW, BW≤1000g) preterm survivors face long-term disabilities such as cerebral palsy, sensory deficits, intellectual impairments, and attention/behavioral problems that significantly impair their quality of life [1]–[3]. Cerebral atrophy/growth disturbances and white matter signal abnormalities (WMSA) are commonly observed following very preterm birth and neonatal intensive care [4]–[8]. These abnormalities, especially when severe, are readily identifiable on conventional T1 or T2 weighted (w) MRI as early as 36 to 40 weeks post-menstrual age [9], [10]. However, qualitative MRI assessments are subjective and prone to measurement errors. Absolute quantification of MRI brain volumes may overcome this limitation for improving neurodevelopmental outcome prediction [11], [12], [13] and qualify as a surrogate endpoint for clinical trials in high risk newborns [13], [14]. To achieve this goal, segmentation methodology needs to be accurate, reliable, and fast. Several investigators have achieved this goal for adult brain MRI segmentation. Most methods classify each voxel in the MRI based on intensity information, spatial information, or a combination of both [15]. Statistical techniques such as expectation-maximization algorithm [16], [17], hidden Markov random field [18], k-nearest neighbor classification [19], [20], and Parzen-window classification [21] have been previously used to correctly identify tissue classes. These novel approaches have facilitated comprehensive and accurate segmentation of adult brain MRI.

In contrast with adults, neonatal brain MRI exhibits lower image contrast due to incomplete myelination, lower signal-to-noise ratio as a result of shorter scan times, and lower spatial resolution due to smaller head size. Segmentation difficulties are further amplified in extremely preterm infants, who exhibit high rates of brain injuries and delayed development. Therefore, automated segmentation of smaller brain structures has been unable to replace manual segmentation methods. Manual approaches, while more accurate, require further improvements in reliability and efficiency for routine use [13], [22], [23]. Particularly, higher segmentation reliability than we previously reported is required for amygdalae, hippocampi, thalamic, and caudate nuclei [13] and reduction in total segmentation time from several days to a few hours is needed to facilitate larger studies [22]. Recently there has been encouraging progress in neonatal cerebral tissue segmentation using probabilistic atlases that exploit anatomical knowledge [24]–[27] and by using regional expectation-maximization algorithm to account for spatial variation of tissue intensity [28]. These approaches have achieved very good to excellent accuracy when compared to expert manual tissue segmentations of cerebrospinal fluid (CSF), gray matter (GM), and white matter (WM). Use of a Parzen window classifier, a nonparametric method that does not assume any intensity distribution may additionally improve accuracy for automated newborn tissue segmentation as achieved with adult MRI [21]. Our aims for this study were four-fold: 1) to develop an accurate neonatal tissue segmentation program that requires minimal operator intervention by adapting the methods of Sajja et al. [21]; 2) to improve the efficiency and reliability of our previously described detailed subcortical manual segmentation methods [13], [23]; 3) to seamlessly integrate these complementary approaches; and 4) to evaluate the accuracy, reliability, and efficiency of the combined comprehensive semi-automated approach.

## Methods

### Ethics Statement

The Children's Hospital and University of Texas Medical School at Houston joint Institutional Review Board approved the study. No parental informed consent was required because the study only analyzed de-identified existing patient data.

### Subjects

A random sample of 30 infants were selected from a consecutively imaged cohort of all ELBW infants that were born and admitted to the NICU of Children's Memorial Hermann Hospital between June 2005 to January 2007 and survived to MRI examination prior to discharge or 38 weeks postmenstrual age (PMA). None of the infants had any major congenital anomalies. Ten ELBW infants (3 males/7 females) were randomly selected for comparison of semi-automated with manual segmentation; their median (95% CL) gestational age was 27 (23–29) weeks, birth weight was 777.5 (530.0–949.0) grams, and PMA at MRI scan was 38.1 (36.4–40.1) weeks. An additional 20 infants (11 males/9 females) were randomly selected to assess manual segmentation reliability; their mean (SD) gestational age was 26.3 (2.3) weeks, birth weight was 722.8 (152.0) grams, and median PMA at MRI scan was 38.0 (range: 35.7 to 43.4) weeks.

### MRI

All ELBW survivors from our NICU were clinically screened for brain injury at 38 weeks PMA or earlier if discharge was sooner, using a standardized conventional MRI protocol on a 1.5 Tesla GE-LX scanner. Sequence parameters for the axial PD/T2w scans used for volumetry were: TE 15/175 ms; TR 10000 ms; ETL 16; FOV 18×18 cm; matrix 512×512; slice thickness 2mm; no gap; voxel height 0.36; width 0.36; depth 1.98 mm. All infants were transported to the MRI scanner by an experienced neonatal transport nurse after feeding, swaddling, and placement of silicone ear plugs. Less than 10% of ELBW infants were administered sedation for excessive movement.

### Manual structural segmentation

Axial PD/T2 images were transferred to a Windows workstation for post-processing and imported into Analyze 8.1 software (Biomedical Imaging Resource, Mayo Clinic, Rochester, MN) for structural segmentation and volume rendering. Due to the poor gray - white matter contrast in the developing brain, subcortical structural segmentation in infants was performed manually. Our previously published methods [23], [13] were modified to improve reliability by greater standardization of structural boundary landmarks, guided by detailed knowledge of regional anatomy. The primary anatomical references used were the Haines neuroanatomy atlas [29], Bayer and Altman atlas of human central nervous system development [30] and two online human atlases [31], [32]. Manually guided boundaries were created in the axial plane and reformatted in the sagittal and coronal planes as needed for difficult structures such as the hippocampus. Distinction between left and right hemisphere structures was not made. The following nine structures were manually segmented, proceeding from inferior to superior axial T2w slices: brain stem, cerebellum, amygdalae, hippocampi, corpus callosum, accumbens, caudate, thalamus, and lenticular nuclei.

The brain stem was segmented first starting with the most inferior slice. It was distinguished by its dark intensity surrounded by bright CSF, central location, and anterior placement to the cerebellum and fourth ventricle. Its rostral margins are below the level of the posterior commissure; anterior and lateral border was defined by the darker subthalmic nuclei, medial by the third ventricle, and posterior boundary by the inferior colliculus. The cerebellum was readily distinguishable from the anteriorly placed brain stem and fourth ventricle by its spatial location and signal intensity differences. The inferior boundary of the amygdalae was defined as the dark almond shaped structure that appears anterior to the frontal horns of the lateral ventricles [33]–[35]. Its superior margins were immediately below the thalamus at the level of the mammillary bodies [36]. The hippocampi inferior border was visible as a dark C-shaped structure posterior and medial to the lateral ventricles and appearing on the same level as the amygdalae. Its anterior border was the amygdala, lateral border the lateral ventricle, medial landmark the subarachnoid fluid, and posterior border the parahippocampal gyrus [34], [35], [37], [38]. The hippocampal superior boundary was determined by the presence of the splenium of the corpus callosum and atrium of the lateral ventricle [39]. The splenium of the corpus callosum's inferior border was medial to and at the level of the superior part of the hippocampus. The inferior boundary of the genu of the corpus callosum first appeared at the level of the inferior portion of the lateral ventricle; it was easily distinguished from surrounding tissues by its dark intensity and position above the lateral ventricles. The superior boundary ends when the left and right hemispheres separate.

**Figure 1:**
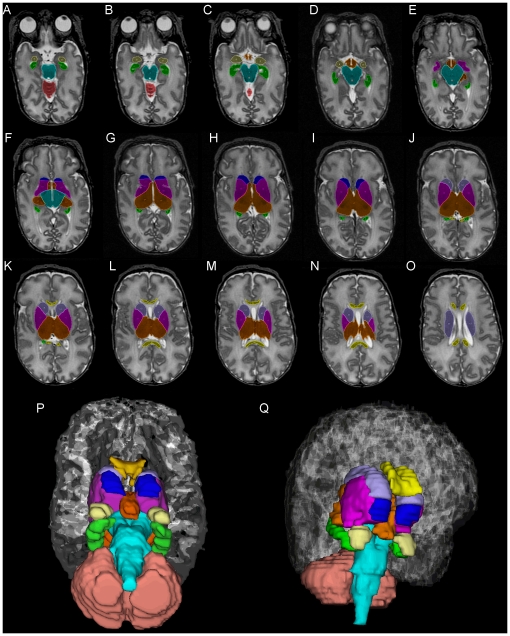
Manually segmented representative axial T2w slices (# 16–31 of 44 total slices) beginning inferiorly with the amygdalae (cream color), hippocampi (green), brain stem (turquoise), cerebellum (copper) (A–C) and progressing superiorly with thalamic (orange), lenticular (pink), accumbens (blue), and caudate nuclei (lavender) and corpus callosum (yellow) (D–O). Final 3-dimensional axial and sagittal oblique models of all nine segmented structures (P and Q).

The inferior border of the nucleus accumbens was at the level of the third ventricle and below the lateral ventricles. Its posterolateral boundary was formed by the anterior limb of the internal capsule and the top of the third ventricle. When the internal capsule was not clearly visible, we extended a horizontal line from the anterior border of the third ventricle to form the posterior border; the remaining borders were readily distinguishable from the surrounding lower intensity white matter. We attempted to isolate the internal capsule but were unable to segment it reliably. Therefore we included this small volume as part of the subcortical structures. The inferior boundary of caudate nucleus starts directly above the accumbens, at the level of the anterior horn of lateral ventricle or subventricular zone [40], [41]. The head of the caudate was bound medially by the frontal horn of the lateral ventricle, laterally by the anterior limb of the internal capsule and posteriorly by the genu of the internal capsule. The caudate superior boundary was above the thalamus and lateral to the confluence of the anterior and posterior horns of the lateral ventricles [42]. The thalamus inferior boundary was defined by the dark centrally located mamillary bodies. Its mid-body was bound by the brain stem posteriorly, posterior limb of the internal capsule laterally, and third ventricle medially. The superior boundary was at the level of the rostral internal capsule; above this, any central gray matter was segmented as caudate nucleus. The lenticular nucleus, comprised of the putamen and globus pallidus, was the only central structure remaining following segmentation of the other subcortical nuclei. It was bound medially by the internal capsule and laterally by the external capsule. A representative example of manually segmented structures is presented in Figure 1.

### Semi-automated tissue segmentation

#### Image pre-processing

Due to lower contrast between CSF/cortex and surrounding extrameningeal tissues (including skull, muscle, and eye structures) compared with adult MRI, extrameningeal tissue stripping (skull stripping) was done semi-automatically in Analyze software with human guidance. Starting from a rater-defined seed point in the bright subarachnoid CSF on one axial T2w slice, the Auto Trace tool was used to threshold the CSF from all the extrameningeal tissues using a region-growing algorithm and copying the setting to the subsequent slices using minor editing as needed. This step took less than 10 minutes per MRI scan.

The skull-stripped images were saved and imported into a workstation for further processing using our in-house developed software under the Interactive Data Language (IDL, Research Systems Inc., Boulder, CO) environment. Anisotropic diffusion filter was applied to reduce the noise without blurring the image [43]. Because the feature maps based on PD/T2w images used for initial classification were generated from a set of training data points, it is essential to normalize intensity distribution of input image volume to that of the training data set. In most cases, this was done automatically by histogram normalization [44] However in one case, automatic histogram normalization was not satisfactory, resulting from excessive motion artifacts. Manual adjustment of the intensity distribution corrected this problem.

#### Automated classification of GM, WM and CSF

We modified the automated segmentation methods of Sajja et al. originally developed for adults with multiple sclerosis [21]. Use of FLAIR images in adults permitted distinction between ventricular CSF (hypointense) and periventricular WM lesions (hyperintense). No such distinction was possible in neonates using FLAIR (both regions appear hypointense). We therefore eliminated the use of FLAIR images and did not attempt to automatically segment WMSA. Based on a training data set of 10 manually segmented ELBW infants' brain MRI scans, a two-dimensional tissue feature map was constructed in the PD-T2 space using Parzen Window classifier with a Gaussian kernel, 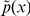 [21], [45], [46]:
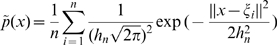
(1)


Here the *n* sample points in the training data set is denoted by *ξ_i_*, *i*∈(1, …., *n*}. Calculation of the parameter, *h_n_* is discussed in detail in our previous publication [21]. Initial classification of GM, WM, and CSF was obtained by classifying each voxel from the input MRI based on its position in the feature map. Then a parametric method, the Hidden Markov Random Field Expectation Maximization algorithm (HMRF-EM), was used to optimize the boundaries between the three tissue clusters in the PD-T2 space. HMRF-EM incorporates contextual information into segmentation through Markov Random Field theory. It is commonly used in research [17], [18], [21], [25], [28] and MRI software (such as FSL and Freesurfer) to capture the spatial homogeneity of tissue classes by favoring assignment of a voxel to the same class of its neighbors. HMRF-EM also corrects for low spatial frequency bias field (or intensity inhomogeneity) as part of segmentation process using EM algorithm. A *d*-dimensional HMRF model with a Gaussian distribution can be specified as:
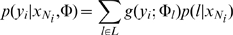
(2)where 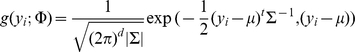 with 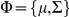.

L represents the set of all class labels, *y_i_* is a feature vector in *d*-dimensional space and 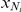 is the neighborhood configuration of 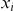 determined from the local characteristics of Markov random fields. Estimation of the model parameters is described elsewhere [18]. Due to partial volume effects, CSF around the brain surface was often misclassified as WM. After the EM step, misclassified WM regions within 2–3 voxels from the brain surface were relabeled as CSF using morphological erosion operation with a 2D kernel measuring 3×3 voxels. This fully automated process took approximately 15 minutes per scan (estimated on an Intel 2.4GHz Core-2 Duo CPU).

### Merging manual structural segmentation and semi-automated tissue segmentation

**Figure 2:**
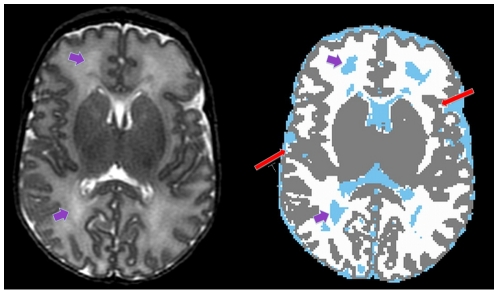
Mid-axial T2w slice on the left highlighting periventricular regions of white matter signal abnormalities (short purple arrows) that the automated segmentation program consistently misclassified as CSF (light blue regions on segmented image on the right). Occasionally subarachnoid CSF was misclassified as WM (long red arrows).

**Figure 3:**
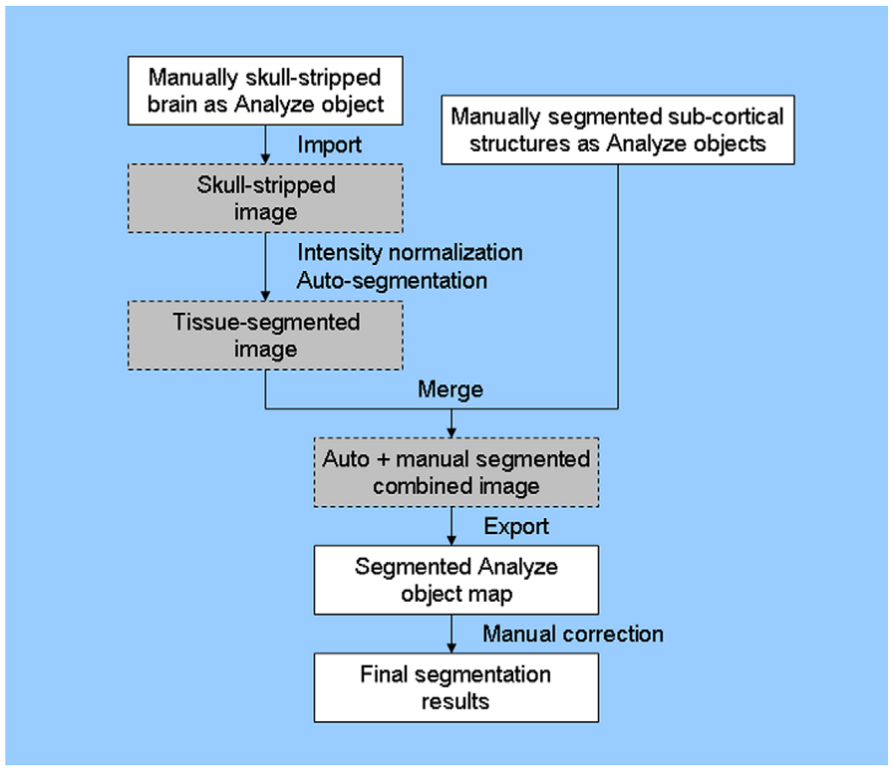
Commercially available Analyze (white boxes) and in-house developed (IDL environment; gray boxes) segmentation programs were integrated seamlessly to permit various preprocessing and segmentation steps. The combined manual structural and automated tissue maps were merged in IDL and exported to Analyze for final manual correction and volume calculations.

The manually generated subcortical segmentation map was imported into IDL and pasted onto the completed automated tissue map. This combined output was then imported into Analyze. To further improve segmentation accuracy, rater one (YZ) inspected the combined map and corrected for any significant tissue classification errors. This rater was trained in neuroanatomy during medical school and in her current job and has been performing detailed manual segmentations for 3 years. The majority of classification errors were mainly observed in the periventricular white matter regions where signal intensities approached that of CSF, resulting in WM being misclassified as CSF (Figure 2). All such misclassified regions were relabeled as WMSA because they always overlapped with areas of T2w abnormalities previously referred to as diffuse excessive high signal intensity [4], [47]. Partial volume effects at the GM-CSF interface also occasionally resulted in misclassification of a few voxels as WM (Figure 2). When significant, these errors were also corrected. It took approximately 25 minutes per MRI scan for manual inspection and correction of the automated tissue segmentation errors. A summary of all the processing steps is provided in Figure 3.

### Evaluating reliability and accuracy

Within-subject standard deviation (SD), repeatability, and intra-class correlation coefficients (ICC) were used to characterize intra- and inter-rater reliability in manual structural segmentation. The within-subject SD is defined as the common SD of repeated measurements and calculated by obtaining the square root of the mean within-subject variance [48]. Repeatability is defined as 2.77 times the within-subject SD. For the same subject, the difference between two measurements is expected to be less than 2.77× within-subject SD for 95% of pairs of observations [48]. Following a rigorous training period, a single trained rater (YZ) manually segmented 20 T2w MRI scans to generate reference volumes for the eight subcortical structures and cerebellum. To assess intra- and inter-rater reliability, all 20 cases were independently segmented again by the same rater and also by a second trained rater (CNG), a minimum of two to four weeks apart, while masked to the initial segmentation results. The one exception was re-segmentation of the cerebellum by the second rater, which was performed in a subset of 10 rather than all 20 cases. Separate random sample of 10 MRI scans were selected for tissue segmentations of cerebral GM, cerebral WM, and CSF (ventricular and subarachnoid). All 10 scans were manually segmented by the first rater. These results served as our reference “gold standard” volumes that were used to assess the accuracy of the IDL semi-automated tissue segmentation program. All 10 cases were independently re-segmented by both raters a minimum of two to four weeks later to assess intra- and inter-rater reliability. We also tested the intra-rater reliability of relabeling WM/CSF misclassification errors as WMSA (N = 10). Both raters were extensively trained and their results independently evaluated by an investigator (NAP) with more than six years experience in performing detailed manual segmentations.

To evaluate automated and semi-automated tissue segmentation against the reference manual parcellation, we used a test data set of 10 MRI (fully independent data set from the training data set used for generating the feature map). Accuracy was assessed using four indices: Dice similarity index (SI) [49], correct estimation index (CEI), over estimation index (OEI), and under estimation index (UEI) [50]. SI measures agreement between the reference manual (Ref) and semi-automated segmentation result (Auto) by calculating the number of voxels that intersect or overlap (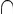) relative to the total number of segmented voxels in both files and is therefore sensitive to differences in size and location [50]. The factor of 2 ensures an SI value of 1 for perfectly matched segmentations. It is the proportion of correctly classified voxels and used as the primary measure of segmentation performance:
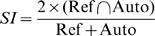
(3a)


Another way of defining SI includes explicit determination of true positives (TP), false positives (FP) and false negatives (FN):
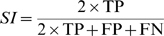
(3b)


CEI measures the ratio of correctly classified voxels relative to the reference:
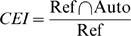
(4a)


OEI measures the ratio of false positive classified voxels relative to the reference while UEI measures the ratio of false negative classified tissues relative to the reference:
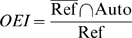
(4b)

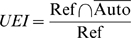
(4c)


CEI, OEI, and UEI provide additional insight into the performance of the segmentation algorithm.

### Statistical analysis

Intra-class correlation coefficient estimates are based on mean squares obtained by applying analysis of variance models to the data using SPSS (Standard Windows Version 10.0.7; Chicago, IL). Within-subject SD, repeatability, SI, CEI, OEI, and UEI as described above, were calculated using IDL and Microsoft Excel (2007 version; Redmond, WA).

## Results

Table 1 presents the mean volumes with 95% confidence limits (CL) for the eight subcortical structures and cerebellum segmented manually twice by the first rater in 20 ELBW infants. Of these, 13 infants were clinically diagnosed with mild to moderate abnormalities, one with severe, and six with no abnormalities on conventional MRI by a neuroradiologist. Table 2 presents three measures of intra-rater segmentation reliability for the first rater. All regions were segmented with high degree of repeatability/reliability, including cerebral GM, cerebral WM, and CSF tissues that served as our reference measurements for assessments of semi-automated segmentation accuracy. Table 3 summarizes inter-rater reliabilities between the first and second raters. While inter-rater ICC values were comparable to intra-rater ICC, repeatability was lower for some regions, particularly for cerebral GM, cerebral WM, and CSF. The standard deviations of tissue and structural volumes were unrelated to their magnitude. White matter signal abnormalities were relabeled with high reliability (ICC 0.999 [95% CI: 0.999 to 1.000]; mean volume: 5524.2 mm^3^; within-subject SD: 42.7 mm^3^; repeatability: 118.3 mm^3^).

**Table 1:** Mean volumes and 95% confidence limits (CL) of manually segmented structures in 20 high risk ELBW infants studied at 38 weeks PMA.

Structures	Mean Volume95% CL (mm^3^)
Cerebellum	15800.9(14215.5, 17386.4)
Brain stem	5603.9(5322.6, 5885.3)
Amygdalae	485.1(445.6, 524.6)
Hippocampi	1211.8(1125.6, 1298.0)
Accumbens	389.2(349.3, 429.1)
Caudate nuclei	2597.0(2391.4, 2802.7)
Lenticular nuclei	5253.1(4931.3, 5574.9)
Thalamus	7248.7(6986.0, 7511.5)
Corpus callosum	803.0(719.7, 886.2)

**Table 2:** *Intra*-rater reliability of manually segmented cerebral structures and tissues volumes.

	Within-subject SD (mm^3^)	Repeatability (mm^3^)	Intra-class correlation coefficient (95% CI)
Cerebellum	261.9	725.4	0.998 (0.994, 0.999)
Brain stem	82.7	229.0	0.990 (0.975, 0.996)
Amygdalae	20.8	57.6	0.970 (0.925, 0.988)
Hippocampi	37.6	104.1	0.981 (0.952, 0.992)
Accumbens nuclei	17.4	48.3	0.984 (0.960, 0.994)
Caudate nuclei	97.1	269.0	0.975 (0.937, 0.990)
Lenticular nuclei	133.9	370.9	0.985 (0.962, 0.994)
Thalamus	134.7	373.0	0.970 (0.925, 0.998)
Corpus callosum	25.0	69.3	0.990 (0.974, 0.996)
Cerebral gray matter	2674.6	7408.5	0.997 (0.988, 0.999)
Cerebral white matter	2633.9	7295.9	0.995 (0.979, 0.999)
Cerebrospinal fluid	1249.4	3460.9	0.997 (0.988, 0.999)

**Table 3:** *Inter*-rater reliability of manually segmented cerebral structures and tissues volumes.

	Within-subject SD (mm^3^)	Repeatability (mm^3^)	Intra-class correlation coefficient (95% CI)
Cerebellum	393.1	1089.0	0.996 (0.983, 0.999)
Brain stem	154.5	427.8	0.974 (0.934, 0.990)
Amygdalae	51.7	143.2	0.942 (0.854, 0.977)
Hippocampi	81.7	226.4	0.970 (0.924, 0.988)
Accumbens nuclei	23.2	64.1	0.984 (0.939, 0.996)
Caudate nuclei	163.0	451.4	0.975 (0.907, 0.993)
Lenticular nuclei	246.8	683.5	0.993 (0.975, 0.998)
Thalamus	166.7	461.8	0.992 (0.972, 0.998)
Corpus callosum	73.7	204.1	0.963 (0.908, 0.986)
Cerebral gray matter	7453.6	20646.3	0.933 (0.732, 0.984)
Cerebral white matter	8402.6	23275.2	0.977 (0.905, 0.994)
Cerebrospinal fluid	1927.3	5338.7	0.998 (0.990, 0.999)

Of the 10 new ELBW infants' MRI scans used to determine tissue segmentation accuracy, seven were diagnosed with mild to moderate abnormalities, one with severe, and two had no reported abnormalities. The mean tissue volumes with 95% CL as determined by the three segmentation approaches are presented in Table 4. Volumes determined by the automated program exhibited minimal differences from manually segmented volumes. The semi-automated approach that permits final correction, further reduced volume differences to 0.2% to 1.5%.

**Table 4:** Mean tissue volumes and 95% CL (mm^3^) using automated, semi-automated, and the reference manually segmented approaches in 10 high risk ELBW infants studied at 38 weeks PMA.

	Automated segmentation	Semi-automated segmentation	Manual segmentation
Cerebral gray matter	94279(87426, 10132)	92973(85758, 100188)	91918(83725, 100111)
Cerebral white matter	113326(102874, 123778)	111753(100731, 122775)	113444(102990, 123899)
Cerebrospinal fluid	62233(53266, 71199)	65239(52872, 77605)	65095(53187, 77003)
Total tissue	269838(247491, 292185)	269965(249104, 290825)	270457(249840, 291075)

**Figure 4:**
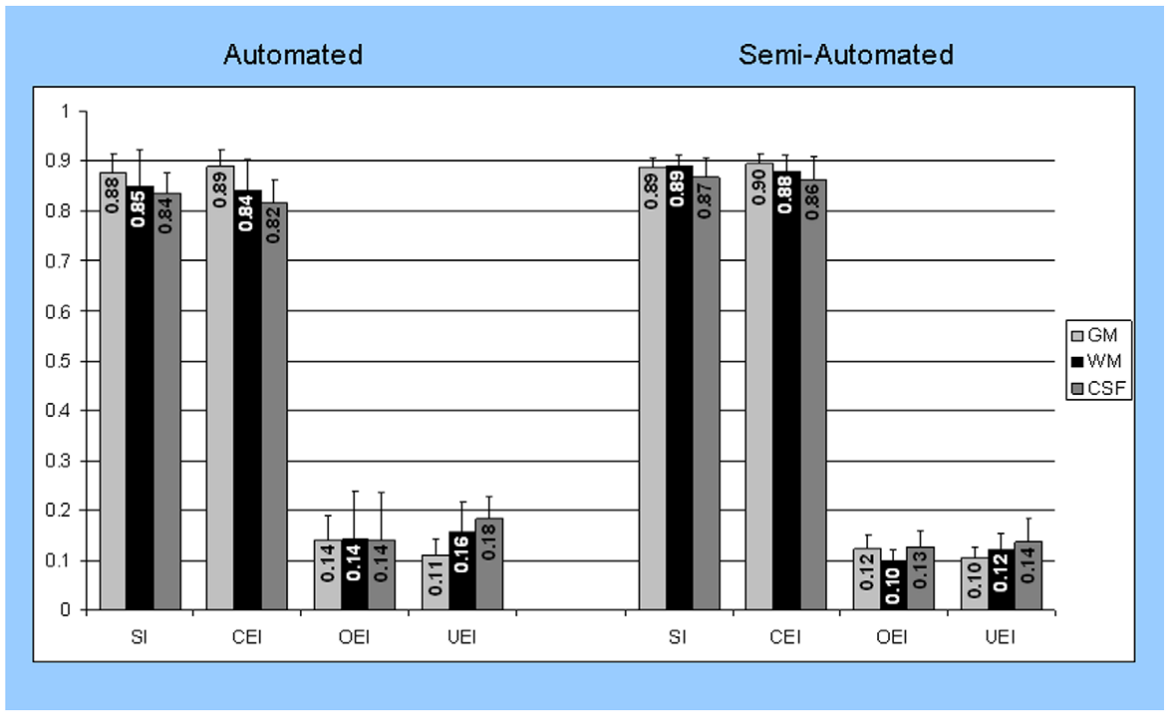
Mean and standard deviations of automated (left) and semi-automated (right) segmentation accuracy and bias measures, including similarity index (SI), correct estimation index (CEI), over estimation index (OEI), and under estimation index (UEI) for cerebral gray matter (light gray), cerebral white matter (black), and CSF (gray).

Figure 4 depicts four measures of accuracy for the automated and the semi-automated segmentation methods. The fully automated approach achieved SI coefficients between 0.84 and 0.88 and correct classification between 0.82 and 0.89 when compared to the reference manual tissue segmentation results. This was a considerable improvement over the first generation of this program that overestimated GM (data not shown). The semi-automated method that includes some manual correction, further improved the SI by about 1–4%, particularly for CSF and WM. Correction of WM/CSF misclassification was primarily required around tissue boundaries (partial volume effects) and in infants with WMSA.

**Figure 5:**
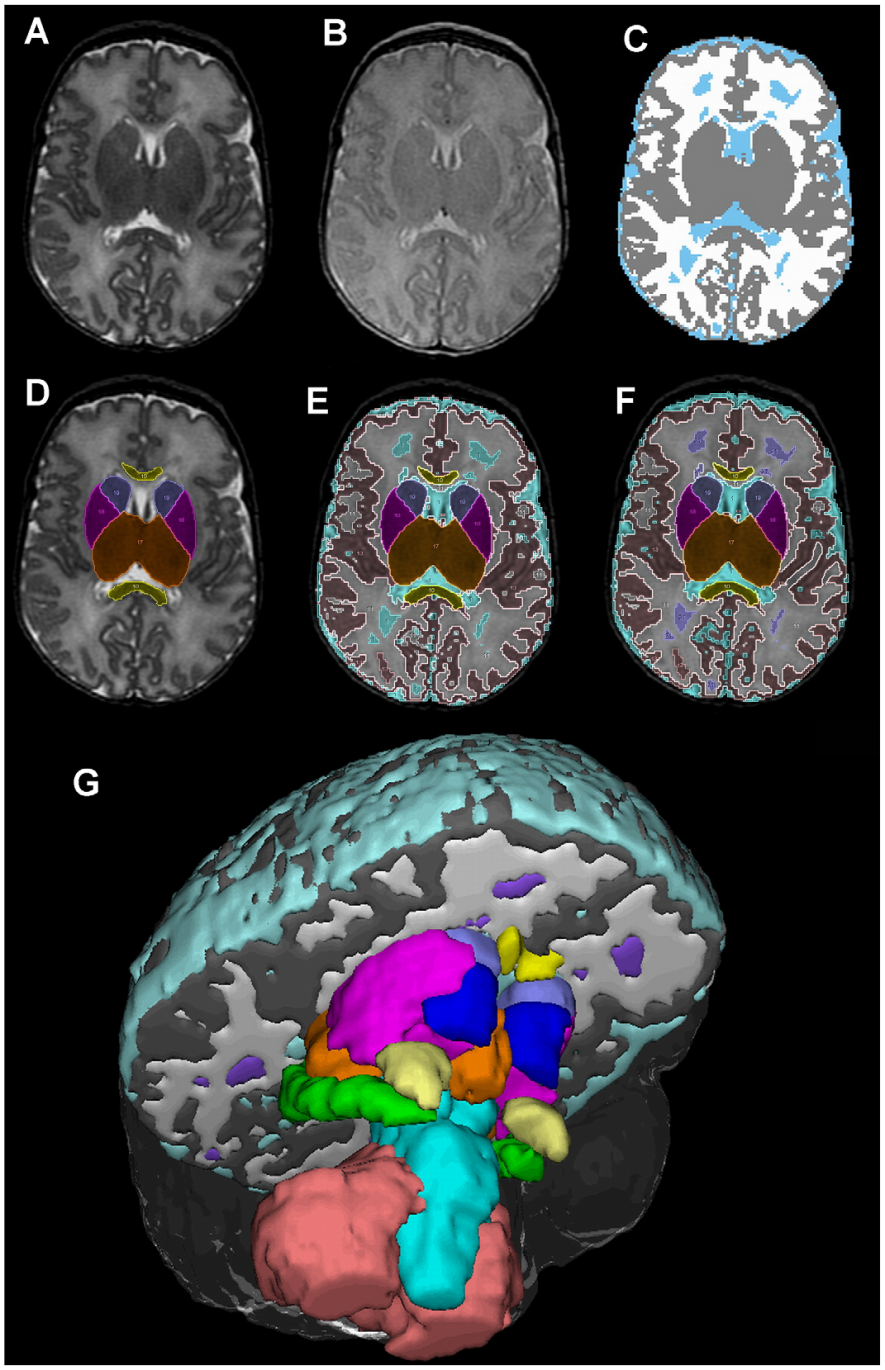
A single mid-axial slice exemplifies results at various stages of processing, beginning with unsegmented conventional axial T2 (A) and proton density weighted images (B), automated three tissue segmentation of cerebral GM (gray), cerebral WM (white), and CSF (light blue) (C), manual structural segmentation output (D), combined automated and manual map without correction (E), and final map following manual correction, including relabeling of WM hyperintensities as WMSA (purple) (F). A 3-dimensional rendering at the same midbrain level displays the relationship of all 13 segmented regions (G).

Using this unified semi-automated approach, each MRI scan took just under two hours to segment into the 13 defined structures and tissue classes. On average, it took the first rater 60 minutes for manual segmentation of the nine structures, 10 minutes for pre-processing for automated segmentation, 15 minutes for automated segmentation of the tissue classes (PC time only), and 25 minutes for final manual correction. Figure 5 displays results from our unified segmentation approach at various stages of processing. The end result, a combination of manual and automated segmentations, generates volumes of nine structures and four tissue classes.

## Discussion

We present a comprehensive and efficient approach to regional brain volume measurements in high risk newborns using reliable manual and accurate semi-automated segmentation methods. This builds on our previously reported methods for detailed structural and tissue segmentation that relied heavily on manual segmentation rendering it less reliable and efficient [13], [23]. These results support our primary aim of developing an accurate, reliable, comprehensive, and efficient approach to MRI brain volume segmentation suitable for further use in large randomized trials or population based studies. Such studies may validate initial reports of the utility of early MRI derived regional volumes as biomarkers of perinatal brain injury [6] and surrogate measures of neurodevelopmental outcomes [11], [12], [51]. Total or regional volumes also appear promising in accurately assessing short-term efficacy and toxicity to neonatal interventions [13], [14], [23], [52]. The current use of pre-discharge cranial ultrasound for these purposes, while efficient and less expensive, lacks sensitivity and reliability [53]–[57]. Accurate and objective quantitative assessments such as regional brain volumes should overcome these limitations. Furthermore, such quantitative outcomes, especially when measured precisely, can, dramatically reduce study sample size needs and facilitate timely assessment of neuroprotective interventions [58]. However, adequately powered qualification studies that evaluate the correlation of regional cerebral volumes with specific neonatal diseases and neurodevelopmental outcomes are required to determine their value as biomarkers and surrogate endpoints [51].

The difficulty of performing fully automated segmentation of the newborn brain has been previously described [19], [24], [26], [28]. Initial efforts in newborns achieved limited accuracy as compared to manual tissue segmentation [19], [24], [59]. Therefore, we had focused our early efforts on developing highly reliable manual segmentations. This culminated in a detailed approach to manual tissue and structural segmentation with high intra-rater ICC, despite the use of previously collected clinical anatomic MRI scans [13], [23]. With this current project we aimed to further improve the reliability by using prospectively standardized MRI scans and more rigorously defined anatomic landmarks. This produced higher intra-rater correlations (all ICCs>0.97) than our prior methods and achieved excellent repeatability. As compared to intra-rater repeatability, there was greater inter-rater variability for manually segmented structures (<2 fold) and tissue classes (up to 3 fold). This lends support for developing and utilizing an automated tissue segmentation program. Visual distinction of smaller cerebral structures is more difficult in newborn MRI scans due to the lower contrast, smaller volumes, and lower signal to noise ratio than in older children. This likely accounts for the paucity of such manual segmentation studies in newborns [22], [60]–[62]. Two studies reported manual segmentation of the hippocampi [60], [62] and one segmented thalamic and lenticular nuclei [61]. Nishida et al. [22] employed a semi-automated technique to additionally segment the cerebellum, brain stem, and amygdalae but not subcortical GM structures, using a convenience sample of 6 to 8 newborns. The number of regions they segmented was comparable to our approach but required prohibitively long processing times (7 days). Compared to our study, these four studies [22], [60], [61], [62] reported similar or slightly lower intra-rater ICC values. However, they did not report intra-rater repeatability or any measures of inter-rater reliability. Bland and Altman have demonstrated that evaluating ICC alone can be misleading and argued for the use of within-subject SD and repeatability as more robust measures of reliability [48], [63]. This is exemplified in our study by discrepant intra-rater and inter-rater repeatability for amygdalae and corpus callosum segmentations, despite similarly high ICC values. Repeatability is defined as the 95% interval for change between two or more repeat measurements. It is more clinically meaningful because a measurement difference that exceeds this value is unlikely to result from measurement error and more reflective of a true clinical change.

Manual segmentations of cerebral GM, cerebral WM, and CSF tissues are more time-consuming and less reliable than subcortical structural segmentations. Therefore, we adapted the adult brain automated segmentation program of Sajja et al. [21] for use in newborns. This innovative approach combines the HMRF-EM parametric approach with the nonparametric Parzen window classifier facilitating robust classification of tissues with a well-defined Gaussian distribution (WM and GM) and those exhibiting skewed distributions (CSF and WMSA). Despite the use of a neonatal training set, initial efforts using this unified approach were met with modest success only. It tended to overestimate the GM and underestimate WM. By implementing flexible intensity normalization, we corrected this bias in class estimation, allowing boundary correction on a per case basis. This resulted in tissue volumes that were comparable to manual volumetry and higher GM and comparable WM and CSF classification accuracy than previous neonatal methods that reported accuracy [26]–[28]. Weisenfeld and Warfield [27] used an atlas based spatial prior approach to additionally segment subcortical GM and myelinated WM with excellent accuracy. Anbeek et al. [26] employed the k-nearest neighbor non-parametric classifier on a 5-dimension feature space that includes 3 spatial dimensions and achieved equally high CSF and subcortical GM accuracy. Similar to other published studies in newborns, they used a convenience sample of more mature low-risk preterm infants without brain abnormalities on MRI or ultrasound. Furthermore, images were likely free of motion artifact, a common problem in newborns, that was overcome by the use of sedation/paralysis or exclusion of such cases. As such, segmentation accuracy may decline in studies of high risk newborns, our target population of interest. Most programs also require the additional acquisition of 3D T1w or inversion recovery sequences that are not routinely obtained during diagnostic MRI. Their use also requires image registration, increasing the likelihood of misclassification errors. Addition of 3D T1w or inversion recovery sequences did not improve the segmentation accuracy of our approach. The sole use of T2/PD sequences, routinely included in clinical studies, permits ready translation of the proposed approach to large cohort studies or randomized trials. The addition of manual volumetry of vulnerable structures yields a final approach that combines the best of automatic segmentation (speed and reproducibility) with manual parcellation (accuracy). With sufficient training, different raters can reliably learn this standardized approach. With approximately 60 minutes of operator time, we were able to segment eight vulnerable subcortical structures and the cerebellum. After another 60 minutes to complete the semi-automated tissue segmentation, full brain segmentation can be accomplished in two hours, a duration that compares favorably to other published methods (1.25 hours [27] to 7 days [22]) and is acceptable for use in large studies.

A few limitations of the proposed methodology deserve consideration. The fully automated segmentation algorithm underestimated CSF by 20%, likely from partial volume averaging, usually resulting in misclassification as WM. While the unified approach permits correction of these errors at the final manual editing stage, efficiency and reliability were slightly reduced. An approach using Markov random field priors to automatically reduce these errors, as reported by Xue et al. [28], may overcome this limitation. Use of manual structural segmentation, while more accurate and still highly reliable, is tedious, time-consuming, and less reproducible than automated methods. Automating this process therefore is an ongoing goal of our work. An additional challenge has been the misclassification of periventricular WM diffuse high signal intensities as CSF owing to its signal intensity overlap with CSF. Because all such misclassification errors occurred in regions that exhibit WMSA, we viewed this limitation as an opportunity and manually relabeled these regions as WMSA. Although this was done relatively objectively, the utility of this approach will remain unknown until such volumes are related to impairments, as we are currently performing. This abnormality has been hypothesized to be a diffuse form of WM injury [8], [47] and associated with lower developmental quotient [10]. However, qualitative assessment of WMSA is highly subjective with low rates of intra- and inter-rater reliability [64]. We observed and reliably quantified WMSA in 90% of our study infants.

Several cohort studies of varying size and duration have examined the correlation of regional newborn brain volumes at term with later neurodevelopmental disabilities [6], [11], [60], [62], [65], [66], [67]. A large majority of these investigators found correlations with motor, cognitive, or sensory deficits measured at 1 to 2 years of age. Lind et al. also reported correlations between total brain and cerebellar volume at term with executive function and motor skills at 5 years of age [68]. Additionally, multiple studies have reported significant correlations between regional brain volumes in adolescent preterm survivors and behavioral, psychological, and cognitive outcomes. These findings reveal the close link between brain atrophy/growth failure and neurodevelopmental impairments and their high incidence following preterm birth and neonatal intensive care. Furthermore, several perinatal risk factors, particularly lower gestational age, intraventricular hemorrhage, white matter injury, and use of postnatal dexamethasone significantly correlate with adverse brain volumes at term [6], [23], [52], [62], [66]. Total brain tissue volume on MRI is also tightly correlated with head circumference [69]. The use of complementary advanced quantitative MRI tools such as diffusion tensor imaging or magnetic resonance spectroscopy may further enhance diagnosis and prediction. Such diagnostic tools may improve parental discharge counseling and permit targeted selection of high risk infants for early intervention studies. School-age neurobehavioral assessments and studies that examine the independent utility of volumetric MRI over conventional MRI and clinical risk factors are however needed to determine the unique value of measuring brain volumes for outcome prediction and risk stratification.

In conclusion, we have developed a reliable, accurate, and efficient semi-automated MRI segmentation approach for detailed brain volume measurements in high risk newborns. This method will facilitate the evaluation of large cohorts to rigorously evaluate the utility of regional brain volumes as biomarkers of neonatal care and surrogate endpoints for neurodevelopmental outcomes.
